# Supplementary material for: Identification of a Kitaev quantum spin liquid by magnetic field angle dependence
Source: Nat Commun. 2022 Jan 14;13:323. doi: 10.1038/s41467-021-27943-9 (PMC8760334; doi:10.1038/s41467-021-27943-9)
Supplement: Supplementary file 1 — Supplementary Information [file 41467_2021_27943_MOESM1_ESM.pdf]

Supplementary Information on "Identification of a Kitaev Quantum Spin Liquid by Magnetic Field Angle Dependence"

Kyusung Hwang,[1,*] Ara Go,[2,3,*] Ji Heon Seong,[4] Takasada Shibauchi,[5] and Eun-Gook Moon[4,†]

[1] *School of Physics, Korea Institute for Advanced Study (KIAS), Seoul 02455, Korea*
[2] *Center for Theoretical Physics of Complex Systems, Institute for Basic Science (IBS), Daejeon 34126, Korea*
[3] *Department of Physics, Chonnam National University, Gwangju 61186, Korea*
[4] *Department of Physics, Korea Advanced Institute of Science and Technology (KAIST), Daejeon 34141, Korea*
[5] *Department of Advanced Materials Science, University of Tokyo, Kashiwa, Chiba 277-8561, Japan*

(Dated: December 27, 2021)

Supplementay Note 1. Magnetic field in various coordinate systems

We use two different coordinate systems defined by $\{\hat{x},\hat{y},\hat{z}\}$ and $\{\hat{a},\hat{b},\hat{c}\}$ for magnetic field:

$$\mathbf{h} = h_x\hat{x} + h_y\hat{y} + h_z\hat{z} = h_a\hat{a} + h_b\hat{b} + h_c\hat{c}. \quad (1)$$

The axes $\{\hat{x},\hat{y},\hat{z}\}$ are the cubic axes of the octahedra enclosing the honeycomb lattice. The other axes $\{\hat{a} \equiv \frac{1}{\sqrt{6}}(\hat{x}+\hat{y}-2\hat{z}), \hat{b} \equiv \frac{1}{\sqrt{2}}(-\hat{x}+\hat{y}), \hat{c} \equiv \frac{1}{\sqrt{3}}(\hat{x}+\hat{y}+\hat{z})\}$ correspond to the bond-perpendicular, bond-parallel, and out-of-plane axes of the honeycomb lattice (Fig. 1a). In the spherical coordinate system (h,θ,ϕ), the magnetic field is written as

$$h_a = h\sin\theta\cos\phi,\ h_b = h\sin\theta\sin\phi,\ h_c = h\cos\theta, \quad (2)$$

and

$$h_x = h\left(-\frac{1}{\sqrt{2}}\sin\theta\sin\phi + \frac{1}{\sqrt{6}}\sin\theta\cos\phi + \frac{1}{\sqrt{3}}\cos\theta\right), \quad (3)$$

$$h_y = h\left(\frac{1}{\sqrt{2}}\sin\theta\sin\phi + \frac{1}{\sqrt{6}}\sin\theta\cos\phi + \frac{1}{\sqrt{3}}\cos\theta\right), \quad (4)$$

$$h_z = h\left(-\sqrt{\frac{2}{3}}\sin\theta\cos\phi + \frac{1}{\sqrt{3}}\cos\theta\right). \quad (5)$$

The polar and azimuthal angles (θ,ϕ) are measured from the out-of-plane axis $\hat{c}$ and the bond-perpendicular axis $\hat{a}$, respectively (Fig. 1b).

Operation	Field-angle transformation
Time reversal	$H(\theta,\phi) \to H(180^\circ-\theta, 180^\circ+\phi)$
Spatial Inversion	$H(\theta,\phi) \to H(\theta,\phi)$
C_3 rotation	$H(\theta,\phi) \to H(\theta, 120^\circ+\phi)$
C_2 rotation (about z-bond)	$H(\theta,\phi) \to H(180^\circ-\theta, 180^\circ-\phi)$

Supplementary Table 1. Symmetry operations of $H_{JK\Gamma\Gamma'}$ and the associated field-angle transformation rules on $H(\theta,\phi)$.

Supplementay Note 2. Symmetry

The symmetries of $H_{JK\Gamma\Gamma'}$ are as follows.

- *Time reversal* : $\mathbf{S}_i \to -\mathbf{S}_i$.
- *Spatial inversion* about each bond center of the honeycomb lattice : $\mathbf{S}_i \to \mathbf{S}_{i'}$.
- *C_3 rotation* about the normal axis to each hexagon plaquette : $(S_i^x, S_i^y, S_i^z) \to (S_{i'}^z, S_{i'}^x, S_{i'}^y)$.
- *C_2 rotation* about each bond axis : $(S_i^x, S_i^y, S_i^z) \to (-S_{i'}^y, -S_{i'}^x, -S_{i'}^z)$ for z-bond rotations.

See Fig. 1a for visualizations of the C_3 and C_2 rotation axes. Transformation rules of $H(\theta,\phi)$ under the symmetry operations are provided in Supplementary Table 1.

Supplementay Note 3. Coupling between the chirality operator and magnetic field

The full expression of the chirality operator χ_p is given by

$$\begin{aligned}\hat{\chi}_p = &S_2^x S_1^z S_6^y + S_5^x S_4^z S_3^y \\ &+ S_6^z S_5^y S_4^x + S_3^z S_2^y S_1^x \\ &+ S_4^y S_3^x S_2^z + S_1^y S_6^x S_5^z\end{aligned} \quad (6)$$

with the site convention shown in Fig. 1a.

We determine the most generic coupling form between the chirality operator and magnetic field using symmetry. Suppose we have $f(\mathbf{h})S_2^x S_1^z S_6^y$ at neighboring three sites 2-1-6 in Fig. 1a. Then $f(\mathbf{h})$ should be a polynomial consisting of only odd powers of $h_{x,y,z}$ by time reversal. C_2 rotation (about the z-bond axis passing through the

* These authors contributed equally: Kyusung Hwang, Ara Go.
† egmoon@kaist.ac.kr

site 1) further constrains $f(\mathbf{h})$ into the form

$$\begin{aligned} & f_{xy,z}(\mathbf{h}) \\ & = \lambda_1(h_x+h_y) + \lambda_2 h_z + \lambda_3 h_x h_y h_z \\ & + \lambda_4(h_x^3+h_y^3) + \lambda_5 h_z^3 + \lambda_6(h_x^2 h_y + h_x h_y^2) \\ & + \lambda_7(h_x^2 h_z + h_y^2 h_z) + \lambda_8(h_x h_z^2 + h_y h_z^2) + \mathcal{O}(h^5). \end{aligned} \tag{7}$$

Lastly, we apply C_3 rotation and spatial inversion to $f_{xy,z}(\mathbf{h}) S_2^x S_1^z S_6^y$, which generates symmetry-related other five terms at a hexagon plaquette:

$$\begin{aligned} & f_{xy,z}(\mathbf{h})(S_2^x S_1^z S_6^y + S_5^x S_4^z S_3^y) \\ & + f_{zx,y}(\mathbf{h})(S_6^z S_5^y S_4^x + S_3^z S_2^y S_1^x) \\ & + f_{yz,x}(\mathbf{h})(S_4^y S_3^x S_2^z + S_1^y S_6^x S_5^z), \end{aligned} \tag{8}$$

where $f_{yz,x}$ and $f_{zx,y}$ are obtained by cyclic permutations of $h_{x,y,z}$ in $f_{xy,z}$. Summing the contributions from all hexagon plaquettes, we arrive at the final expression

$$\sum_{\langle ij\rangle_\alpha \langle jk\rangle_\beta} f_{\alpha\beta,\gamma}(\mathbf{h}) S_i^\alpha S_j^\gamma S_k^\beta. \tag{9}$$

These effective interactions are generated by applied magnetic fields, and the coupling functions $f_{\alpha\beta,\gamma}(\mathbf{h})$ can be evaluated by the perturbation theory shown below.

Our major finding is summarized before the construction of the perturbation theory. The coupling functions $f_{\alpha\beta,\gamma}(\mathbf{h})$ determine topological properties of the non-abelian Kitaev quantum spin liquid (KQSL). In particular, the chirality is given by

$$\chi = \frac{1}{N}\sum_p \langle \hat{\chi}_p \rangle \approx -[f_{xy,z}(\mathbf{h}) + f_{yz,x}(\mathbf{h}) + f_{zx,y}(\mathbf{h})], \tag{10}$$

and its sign and magnitude are closely related with the Chern number and the energy gap of Majorana fermion excitations. The chirality can be arranged in terms of A_2 representations of $\mathbf{h}$:

$$\chi \approx \Lambda_1 F_1 + \Lambda_3 F_3 + \Lambda_3' F_3' + \Lambda_3'' F_3'' + \mathcal{O}(h^5), \tag{11}$$

where the coefficients (Λ) and the A_2 representations (F) are listed in Supplementary Table 2. The Λ coefficients are obtained by the perturbation theory below.

Supplementay Note 4. Perturbative expansion

The perturbation theory is developed by decomposing the full Hamiltonian H into the unperturbed part ($H_0 = \sum_{\langle jk\rangle_\gamma} K S_j^\gamma S_k^\gamma$) and perturbation part ($H' = H - H_0$). In this setup, we focus on the zero-flux sector of the pure Kitaev model H_0 (*i.e.*, $\langle \hat{W}_p \rangle = 1$).

For a systematic derivation, we employ a quasi-degenerate perturbation theory [1] and construct an effective Hamiltonian $\mathcal{H}$ in the zero-flux sector. Up to the third order of H', the effective Hamiltonian $\mathcal{H}$ is formally given by

$$\begin{aligned} \mathcal{H} = PH_0P &- \frac{1}{\Delta_{\text{flux}}} PH'QH'P \\ &+ \frac{1}{\Delta_{\text{flux}}^2} PH'QH'QH'P \\ &- \frac{1}{2\Delta_{\text{flux}}^2}(PH'PH'QH'P + \text{H.c.}), \end{aligned} \tag{12}$$

where P is the projection operator into the zero-flux sector, and $Q = 1 - P$. Here we have assumed that energy differences between the zero-flux sector and nonzero-flux sectors are simply given by the flux gap $\Delta_{\text{flux}}(= 0.26S^2|K|) = 0.065|K|$ [2]. In order for the perturbation theory to be valid, non-Kitaev couplings should be much smaller than the flux gap ($J, \Gamma, \Gamma', h \ll \Delta_{\text{flux}}$).

The λ coefficients in Supplementary Eq. (7) are obtained by the perturbative calculations of Supplementary Eq. (12), which is demonstrated by using the second order term $-\frac{1}{\Delta_{\text{flux}}} PH'QH'P$ as an example. Among various interactions generated by the perturbation, we collect components of the chirality operator, $S_i^\alpha S_j^\gamma S_k^\beta$, which are defined over three neighboring sites ijk connected by two bonds $\langle ij\rangle_\alpha$ and $\langle jk\rangle_\beta$ with all different α, β, γ. This type of terms are produced by $PH'QH'P$ via two processes: (i) $S_i^\alpha S_j^\gamma$ from the Γ' coupling and S_k^β from the Zeeman coupling, or (ii) S_i^α from the Zeeman coupling and $S_j^\gamma S_k^\beta$ from the Γ' coupling. These second order processes re-

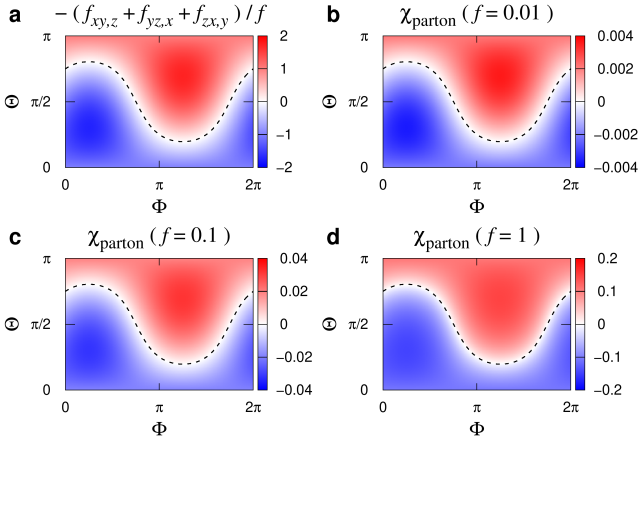

Supplementary Figure 1. Chirality χ_{parton} of the parton theory. a-d Color maps of $-(f_{xy,z} + f_{yz,x} + f_{zx,y})/f$ and χ_{parton} ($f = 0.01, 0.1, 1$) as functions of Θ and Φ. In each plot, the dashed line indicates the points of $f_{xy,z} + f_{yz,x} + f_{zx,y} = 0$.

Coefficient	A_2 representation
$\Lambda_1 = -(2\lambda_1 + \lambda_2)$	$F_1(\mathbf{h}) = h_x + h_y + h_z$
$\Lambda_3 = -3\lambda_3$	$F_3(\mathbf{h}) = h_x h_y h_z$
$\Lambda_3' = -(2\lambda_4 + \lambda_5)$	$F_3'(\mathbf{h}) = h_x^3 + h_y^3 + h_z^3$
$\Lambda_3'' = -(\lambda_6 + \lambda_7 + \lambda_8)$	$F_3''(\mathbf{h}) = h_x^2 h_y + h_x h_y^2 + h_y^2 h_z + h_y h_z^2 + h_z^2 h_x + h_z h_x^2$

Supplementary Table 2. Expansion of the chirality χ in terms of A_2 representations of h.

sult in the effective interaction

$$-\frac{1}{\Delta_{\text{flux}}} P H' Q H' P \tag{13}$$
$$= P\left[\sum_{\langle ij\rangle_\alpha \langle jk\rangle_\beta} \frac{2\Gamma'(h_\alpha + h_\beta)}{\Delta_{\text{flux}}} S_i^\alpha S_j^\gamma S_k^\beta\right] P + \cdots$$

that corresponds to the λ_1 term of Supplementary Eq. (7). Note that the components of the chirality operator appear in the second order because of the presence of Γ' in drastic contrast to the pure Kitaev model where the chirality operator is generated in the third order [3].

Repeating the perturbative expansion up to the third order, we obtain the effective Hamiltonian

$$\mathcal{H} = P\left[H_0 + \sum_{\langle ij\rangle_\alpha \langle jk\rangle_\beta} f_{\alpha\beta,\gamma}(\mathbf{h}) S_i^\alpha S_j^\gamma S_k^\beta\right] P + \cdots, \tag{14}$$

where the f function is given by Supplementary Eq. (7) with the λ coefficients:

$$\lambda_1 = \frac{2\Gamma'}{\Delta_{\text{flux}}} - \frac{5J\Gamma'}{2\Delta_{\text{flux}}^2} + \frac{2\Gamma\Gamma'}{\Delta_{\text{flux}}^2} - \frac{\Gamma'^2}{2\Delta_{\text{flux}}^2}, \tag{15}$$

$$\lambda_2 = -\frac{J\Gamma'}{\Delta_{\text{flux}}^2} - \frac{3\Gamma'^2}{2\Delta_{\text{flux}}^2}, \tag{16}$$

$$\lambda_3 = -\frac{6}{\Delta_{\text{flux}}^2}. \tag{17}$$

The other coefficients $\lambda_{4,5,6,7,8}$ remain zero ($\lambda_{4,5,6,7,8} = 0$) up to third order.

Supplementay Note 5. Parton theory

Energy gap. The component of the chirality operator $S_i^\alpha S_j^\gamma S_k^\beta$ gives rise to a next-nearest-neighbor hopping term of c-Majorana fermions without changing the flux sector [2, 4]. This can be seen from the identity

$$S_i^\alpha S_j^\gamma S_k^\beta = -\frac{i}{8} D_j \hat{u}_{ij} \hat{u}_{kj} c_i c_k, \tag{18}$$

where $\hat{u}_{ij} = i b_i^\alpha b_j^\alpha$, $\hat{u}_{kj} = i b_k^\beta b_j^\beta$, and $D_j = b_j^x b_j^y b_j^z c_j$. Applying this to $\mathcal{H}$ in Supplementary Eq. (14), we obtain the effective Majorana Hamiltonian

$$\mathcal{H} = P\left[\sum_{\langle jk\rangle_\gamma} \frac{-iK}{4} \hat{u}_{jk} c_j c_k + \sum_{\langle ij\rangle_\alpha \langle jk\rangle_\beta} \frac{-i f_{\alpha\beta,\gamma}}{8} D_j \hat{u}_{ij} \hat{u}_{kj} c_i c_k\right] P. \tag{19}$$

For the calculations of fermion excitation spectrum, we choose the uniform configuration of $\mathbb{Z}_2$ gauge fields: $\hat{u}_{jk} = 1$ for $j \in A$, $k \in B$ (or $\hat{u}_{jk} = -1$ for $j \in B$, $k \in A$), where A, B imply two sublattices of the honeycomb lattice. After the gauge-fixing and Fourier transformation to momentum space, the Hamiltonian is written as

$$\mathcal{H} = \frac{1}{4}\sum_{\mathbf{q}} \left[\begin{array}{cc} c_{-\mathbf{q}}^A & c_{-\mathbf{q}}^B \end{array}\right] \left[\begin{array}{cc} M_\mathbf{q} & iU_\mathbf{q} \\ -iU_\mathbf{q}^* & -M_\mathbf{q} \end{array}\right] \left[\begin{array}{c} c_\mathbf{q}^A \\ c_\mathbf{q}^B \end{array}\right], \tag{20}$$

where the subscript $\mathbf{q}$ denotes momentum, and the superscripts A, B imply two sublattices of the honeycomb lattice. The matrix elements are given by $M_\mathbf{q} = -\frac{1}{2}[f_{xy,z} \sin \mathbf{q}\cdot(\mathbf{n}_2 - \mathbf{n}_1) + f_{yz,x} \sin \mathbf{q}\cdot(-\mathbf{n}_2) + f_{zx,y} \sin \mathbf{q}\cdot \mathbf{n}_1]$, and $U_\mathbf{q} = -\frac{K}{2}(1 + e^{i\mathbf{q}\cdot\mathbf{n}_1} + e^{i\mathbf{q}\cdot\mathbf{n}_2})$, where $\mathbf{n}_1$, $\mathbf{n}_2$ imply lattice vectors of the honeycomb lattice. In terms of the complex fermion operators $\Psi_\mathbf{q} = \left[\psi_\mathbf{q}, \psi_{-\mathbf{q}}^\dagger\right]^T \equiv \frac{1}{2}\left[c_\mathbf{q}^A + i c_\mathbf{q}^B, c_\mathbf{q}^A - i c_\mathbf{q}^B\right]^T$, the Hamiltonian takes the form

$$\mathcal{H} = \frac{1}{2}\sum_\mathbf{q} \Psi_\mathbf{q}^\dagger\, \mathbf{R}(\mathbf{q})\cdot\boldsymbol{\sigma}\, \Psi_\mathbf{q}, \tag{21}$$

where $\mathbf{R}(\mathbf{q}) = [M_\mathbf{q}, \text{Im}U_\mathbf{q}, \text{Re}U_\mathbf{q}]$ and $\boldsymbol{\sigma} = [\sigma_x, \sigma_y, \sigma_z]$ are the Pauli matrices. Taking a canonical transformation to the quasiparticle basis $\gamma_\mathbf{q} = u_\mathbf{q}\psi_\mathbf{q} + v_\mathbf{q}\psi_{-\mathbf{q}}^\dagger$, we obtain the diagonalized Hamiltonian $\mathcal{H} = E_{\text{gs}} + \sum_\mathbf{q} \omega_\mathbf{q} \gamma_\mathbf{q}^\dagger \gamma_\mathbf{q}$ with the excitation spectrum $\omega_\mathbf{q} = \sqrt{|M_\mathbf{q}|^2 + |U_\mathbf{q}|^2}$ and the ground state energy $E_{\text{gs}} = -\frac{1}{2}\sum_\mathbf{q} \omega_\mathbf{q}$. The gapless points $\pm\mathbf{q}_* = \pm(\frac{1}{3}\mathbf{q}_1 + \frac{2}{3}\mathbf{q}_2)$ of the pure Kitaev model are gapped out by the energy gap

$$\Delta_{\text{parton}} = \omega_{\mathbf{q}_*} = \frac{\sqrt{3}}{4}|-(f_{xy,z} + f_{yz,x} + f_{zx,y})|, \tag{22}$$

where $\mathbf{q}_{1,2}$ are the reciprocal lattice vectors dual to $\mathbf{n}_{1,2}$.

Chern number. The gapped spin liquid state is topo-

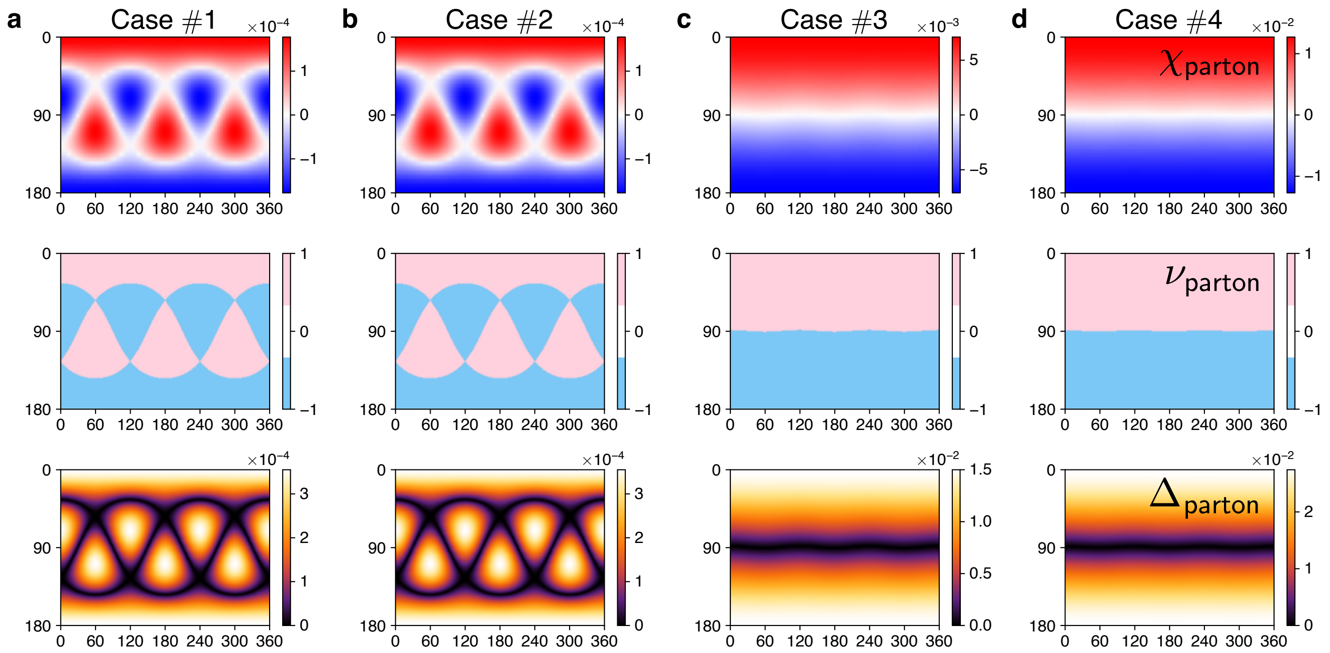

Supplementary Figure 2. **Results of the perturbative parton theory.** Top, middle, bottom: color maps of the chirality $\chi_{\text{parton}}(\mathbf{h})$, the Chern number $\nu_{\text{parton}}(\mathbf{h})$, and the Majorana energy gap $\Delta_{\text{parton}}(\mathbf{h})$ on the plane of the field angles (θ, ϕ), where the magnetic field strength is fixed by $h = 0.01$ (horizontal axis: ϕ [°], vertical axis: θ [°]). The parameter sets used in the four cases (#1 ∼ 4) are listed in Table 1.

logically characterized by the Chern number [2]

$$\nu_{\text{parton}} = \int \frac{d^2\mathbf{q}}{4\pi} [\hat{\mathbf{R}} \cdot \partial_{q_x} \hat{\mathbf{R}} \times \partial_{q_y} \hat{\mathbf{R}}] = \text{sgn}[-(f_{xy,z} + f_{yz,x} + f_{zx,y})] \tag{23}$$

which takes either +1 or −1 depending on the field direction.

Chirality. Finally, we compute the expectation value of the chirality operator $\hat{\chi}$:

$$\begin{aligned}\chi_{\text{parton}} &= \frac{1}{N} \sum_p \langle \hat{\chi}_p \rangle \\ &= \frac{1}{N} \left(\frac{\partial}{\partial f_{xy,z}} + \frac{\partial}{\partial f_{yz,x}} + \frac{\partial}{\partial f_{zx,y}} \right) E_{\text{gs}} \\ &= -\frac{1}{2N} \sum_{\mathbf{q}} \frac{M_{\mathbf{q}} L_{\mathbf{q}}}{\omega_{\mathbf{q}}},\end{aligned} \tag{24}$$

where N is the total number of unit cells, and $L_{\mathbf{q}} = -\frac{1}{2}[\sin \mathbf{q}\cdot(\mathbf{n}_2 - \mathbf{n}_1) + \sin \mathbf{q}\cdot(-\mathbf{n}_2) + \sin \mathbf{q}\cdot\mathbf{n}_1]$. By conducting numerical computations for χ_{parton}, we find the following property:

$$\chi_{\text{parton}} = -(f_{xy,z} + f_{yz,x} + f_{zx,y})\ g, \tag{25}$$

where g is a positive function of $(f_{xy,z}, f_{yz,x}, f_{zx,y})$. In the limit of $f = \sqrt{f_{xy,z}^2 + f_{yz,x}^2 + f_{zx,y}} \to 0$, we find that $g \to 0.21$. Supplementary Fig. 1 illustrates the chirality χ_{parton} with the parametrization

$$f_{yz,x} = f \sin\Theta \cos\Phi, \tag{26}$$

$$f_{zx,y} = f \sin\Theta \sin\Phi, \tag{27}$$

$$f_{xy,z} = f \cos\Theta, \tag{28}$$

where $f > 0$, $\Theta \in [0, \pi]$, and $\Phi \in [0, 2\pi]$. On the plane of Θ and Φ, χ_{parton} exhibits exactly the same pattern of sign structure as that of $-(f_{xy,z} + f_{yz,x} + f_{zx,y})$; compare Supplementary Fig. 1b-d with a. Therefore, we obtain the relationship

$$\chi_{\text{parton}} \propto -(f_{xy,z} + f_{yz,x} + f_{zx,y}). \tag{29}$$

Notice that the chirality itself is intimately related with the Chern number and the Majorana energy gap: $\nu = \text{sgn}(\chi)$ and $\Delta \sim |\chi|$.

Combining with the results of Supplementary Eqs. (15)-(17), we obtain the chirality,

$$\chi_{\text{parton}}(\mathbf{h}) \propto \Lambda_1(h_x + h_y + h_z) + \Lambda_3 h_x h_y h_z, \tag{30}$$

the Chern number,

$$\nu_{\text{parton}}(\mathbf{h}) = \text{sgn}[\Lambda_1(h_x + h_y + h_z) + \Lambda_3 h_x h_y h_z], \tag{31}$$

and the Majorana energy gap,

$$\Delta_{\text{parton}}(\mathbf{h}) \propto |\Lambda_1(h_x+h_y+h_z)+\Lambda_3 h_x h_y h_z|\,, \tag{32}$$

where $\Lambda_1 = -(2\lambda_1+\lambda_2)$ and $\Lambda_3 = -3\lambda_3$. These results are pictorialized in Supplementary Fig. 2.

Supplementay Note 6. Relation between the chirality and Majorana gap

The relation, $|\chi| \propto \Delta$, between the chirality and the Majorana energy gap has been established by using the perturbative parton analysis near the pure Kitaev model [Supplementary Eqs. (30,32)]. Here, we provide our reasoning why the relation is expected to hold more generally in our KQSL phase diagrams.

First, we find that the relation $|\chi| \propto \Delta$ holds near the universal zeroes for a *generic* KQSL with D_3 symmetry. To show this, let us recall that magnetic fields along the bond directions do not break the bond direction C_2 symmetry, which makes both χ and Δ to be zero. Suppose we slightly tilt the magnetic field from a bond direction by a small angle $\delta\phi$ ($\ll 1$). The tilting effects appear in the parton Hamiltonian as

$$\begin{bmatrix} 0 & iU_{\mathbf{q}} \\ -iU^*_{\mathbf{q}} & 0 \end{bmatrix} \xrightarrow{\text{Tilting the field by } \delta\phi} \begin{bmatrix} m\delta\phi & iU_{\mathbf{q}} \\ -iU^*_{\mathbf{q}} & -m\delta\phi \end{bmatrix}. \tag{33}$$

It is obvious that Majorana fermions acquire a mass term ($m\delta\phi$), which is an A_2 representation of the D_3 symmetry. Then, both of χ and Δ are proportional to $\delta\phi$ because they are the same A_2 representation, and the linear relationship, $|\chi| \propto \Delta$, is thus established near the universal zeroes. We stress that the D_3 symmetry plays a significant role in our results. In other words, the universal zeroes always appear in a generic KQSL with the D_3 symmetry, and the relationship must hold in any points of KQSL in our phase diagrams.

Furthermore, we consider another exactly solvable model,

$$H_\chi = K\sum_{\langle jk\rangle_\gamma} S^\gamma_j S^\gamma_k - M\sum_p \hat{\chi}_p, \tag{34}$$

which consists of the Kitaev interactions (K) and the chirality operator three-spin interactions (M). The parton analysis shows that the Chern number is $\nu = \text{sgn}(M)$ and the Majorana gap is $\Delta = \frac{3\sqrt{3}}{4}|M|$. Supplementary Fig. 3 presents the calculated chirality χ as a function of M; overall, χ is proportional to M. The positive correlation between χ and M is demonstrated, establishing the connection between the chirality and the Marjorana gap in a fairly large window ($|M/K| \lesssim 0.5$).

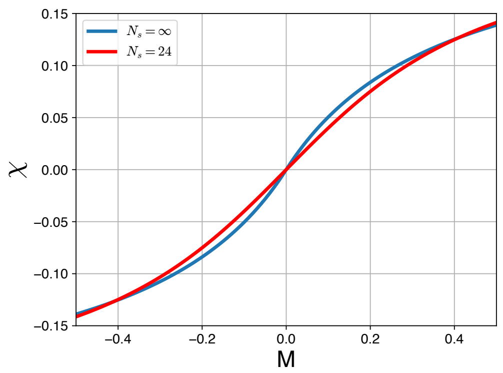

Supplementary Figure 3. Chirality of the model H_χ. The chirality χ as a function of the coupling constant M. Red: ED calculation results for the 24-site cluster. Blue: parton analysis results for the infinite system. In all these results, the Kitaev coupling is fixed by $K=-1$.

Supplementay Note 7. Energy current operator

To derive the energy current operator $\mathbf{J}^{\text{E}}$, we arrange the Hamiltonian [Eq. (1)] as

$$H = \sum_{\langle jk\rangle} H^{\text{EX}}_{jk} + \sum_i H^{\text{Z}}_i, \tag{35}$$

where H^{EX}_{jk} is the exchange interaction at bond $\langle jk\rangle$, and H^{Z}_i is the Zeeman coupling at site i. Then the energy polarization operator can be written as

$$\mathbf{P}^{\text{E}} = \sum_{\langle jk\rangle} \frac{\mathbf{r}_j+\mathbf{r}_k}{2} H^{\text{EX}}_{jk} + \sum_i \mathbf{r}_i H^{\text{Z}}_i, \tag{36}$$

where $\mathbf{r}_i$ means the position vector of site i. The energy current is nothing but the time derivative of the polarization [5, 6]:

$$\begin{aligned}\mathbf{J}^{\text{E}} &= \frac{d\mathbf{P}^{\text{E}}}{dt} = i[H,\mathbf{P}^{\text{E}}] \qquad (37)\\ &= i\sum_{\langle jk\rangle}\sum_{\langle j'k'\rangle}\frac{\mathbf{r}_{j'}+\mathbf{r}_{k'}}{2}[H^{\text{EX}}_{jk},H^{\text{EX}}_{j'k'}] + i\sum_i\sum_{i'}\mathbf{r}_{i'}[H^{\text{Z}}_i,H^{\text{Z}}_{i'}]\\ &+ i\sum_{\langle jk\rangle}\sum_{i'}\mathbf{r}_{i'}[H^{\text{EX}}_{jk},H^{\text{Z}}_{i'}] + i\sum_i\sum_{\langle j'k'\rangle}\frac{\mathbf{r}_{j'}+\mathbf{r}_{k'}}{2}[H^{\text{Z}}_i,H^{\text{EX}}_{j'k'}].\end{aligned}$$

In the last equality, the first (second) line shows time reversal odd (even) terms. Interestingly, time reversal odd terms only arise from the commutator $[H^{\text{EX}}_{jk},H^{\text{EX}}_{j'k'}]$ because the other commutator is alway zero ($[H^{\text{Z}}_i,H^{\text{Z}}_{i'}]=0$). As an example, explicit calculations for the Kitaev limit lead to the current operator

$$\mathbf{J}^{\text{E}} = \sum_p \mathbf{j}^{\text{E}}_p \tag{38}$$

where the local current operator $\mathbf{j}_p^{\rm E}$ at plaquette p is given by

$$\begin{aligned}\mathbf{j}_p^{\rm E} = & \frac{K^2}{2}(\mathbf{r}_2-\mathbf{r}_6)S_2^xS_1^zS_6^y + \frac{K^2}{2}(\mathbf{r}_5-\mathbf{r}_3)S_5^xS_4^zS_3^y \\ & + \frac{K^2}{2}(\mathbf{r}_6-\mathbf{r}_4)S_6^zS_5^yS_4^x + \frac{K^2}{2}(\mathbf{r}_3-\mathbf{r}_1)S_3^zS_2^yS_1^x \\ & + \frac{K^2}{2}(\mathbf{r}_4-\mathbf{r}_2)S_4^yS_3^xS_2^z + \frac{K^2}{2}(\mathbf{r}_1-\mathbf{r}_5)S_1^yS_6^xS_5^z \\ & + \text{(time reversal even terms)}. \end{aligned} \quad (39)$$

Notice the appearance of the components of the chirality operator $\hat{\chi}_p$ [6].

Supplementay Note 8. Validity conditions of the perturbative parton theory and chirality operator method

The perturbative parton theory and chirality operator method become exact in the limit of the pure Kitaev model under weak magnetic field. Yet, it is important to note that the two methods have their own validity conditions. It is exceedingly difficult to prove which one is a better approach in general, and additional careful analysis is necessary if the two methods show discrepancy. Below, we discuss the validity conditions of each method as well as how to improve the discrepancy.

Recall that the topological invariant (ν) is determined by the zero temperature limit of thermal hall conductivity over temperature, κ_{xy}/T. The perturbative parton theory introduces parameters made of non-Kitaev interactions and flux gap such as $\mathit{\Gamma}'/\Delta_{\rm flux}$, and the topological invariant can be written as the expansion,

$$\nu = \mathrm{sgn}\left[m_0 + m_1\left(\frac{\mathit{\Gamma}'}{\Delta_{\rm flux}}\right) + m_2\left(\frac{\mathit{\Gamma}'}{\Delta_{\rm flux}}\right)^2 + \cdots\right].$$

The coefficients $m_{0,1,\cdots}$ are functions of the coupling constants of a given Hamiltonian $(K, J, \mathit{\Gamma}, \mathit{\Gamma}', \cdots)$. The perturbative parton theory is valid if the parameters such as $\mathit{\Gamma}'/\Delta_{\rm flux}$ are small enough. The case #2 is expected to be similar to the case #1 as manifested in Fig. 2. For the cases (#3,4), the perturbation parameters are larger, for example $\mathit{\Gamma}'/\Delta_{\rm flux} = 0.77$. Though the perturbative parton theory is powerful, we also note that the convergence of the expansion is neither guaranteed nor proven, especially with multiple coupling constants.

The chirality operator method, on the other hand, is based on a different type of expansions by exploiting symmetry properties. Since ν is in the time reversal odd A_2 representation of D_3 symmetry, we consider the operator associated with κ_{xy}, commutator/correlator of energy currents as used in Ref. [6], which can be formally written as

$$\hat{A}_2 = M_3 \sum \hat{\chi}_3 + M_5 \sum \hat{\chi}_5 + M_7 \sum \hat{\chi}_7 + \cdots .$$

The string operators with an odd number of spin operators connecting two sites on a same sublattice ($\hat{\chi}_{3,5,7}$) are introduced, whose graphical representations are given in Supplementary Fig. 4. Their expectation values ($\langle\hat{\chi}_{3,5,7}\rangle$) determine ν. Note that there is no linear term in the expansion as shown in Supplementary Note 7, and the first term $\sum \hat{\chi}_3$ is identical to the chirality operator $\sum_p \hat{\chi}_p$. The coefficients $M_{3,5,7}$ are functions of the coupling constants of a given Hamiltonian $(K, J, \mathit{\Gamma}, \mathit{\Gamma}', \cdots)$ while the string operators are independent. The chirality operator method is to truncate the expansion at the leading chirality operator term to determine ν.

The ED calculations with the chirality operator are valid under the conditions,

$$\left|M_3 \sum\langle\hat{\chi}_3\rangle\right| \gg \left|M_5 \sum\langle\hat{\chi}_5\rangle\right|, \left|M_7 \sum\langle\hat{\chi}_7\rangle\right|, \cdots . \quad (40)$$

The conditions hold near the pure Kitaev model as manifested in the perfect matches between the ED calculations and the perturbative parton theory for the cases (#1,2). The structure of the chirality operator method can be further analyzed by evaluating the expectation values of $\hat{\chi}_{3,5,7}$ for the cases (#1-4) with a magnetic field along the [111] direction, shown in Supplementary Fig. 4. We find that the cases (#3,4) have hundred times larger values of $|\langle\hat{\chi}_n\rangle|$ compared to the cases (#1,2), which are consistent with the larger perturbation parameters of the perturbative parton theory. Moreover, we find that the two ratios,

$$r_1 \equiv \frac{|\langle\hat{\chi}_5\rangle|}{|\langle\hat{\chi}_3\rangle|} \sim \frac{1}{10}, \qquad r_2 \equiv \frac{|\langle\hat{\chi}_7\rangle|}{|\langle\hat{\chi}_3\rangle|} \sim \frac{1}{100}$$

for the four cases. Since the chirality operator method works well for the cases (#1,2), the validity conditions [Supplementary Eq. (40)] can be fulfilled if the coefficients $M_{3,5,7}$ of the cases (#3,4) are not much different from the ones of the cases (#1,2).

To check the validity conditions, Supplementary Eq. (40), one needs to calculate the coefficients $M_{3,5,7}$ explicitly. Leaving them for future works, we instead notice that the coefficients $M_{3,5,7}$ are in the trivial representation of the D_3 symmetry, being functions of energy eigenvalues. We calculate the energy spectrum variances of the lowest hundred energy eigenvalues for the four cases and find that the variances are all less than 5%. Thus, it is tempting to assume that $M_{3,5,7,\cdots}$ do not vary much, and then, the ED calculations with the chirality operator would work.

Our proposal with the chirality operator is not only alternative but also complementary to the perturbative parton analysis. Namely, the perfect matches between the two methods' results for the cases (#1,2) become a sanity check as shown in Fig. 2. If the two methods show

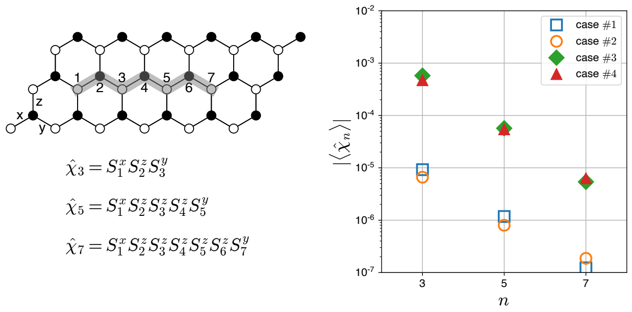

Supplementary Figure 4. **Expectation values of the** A_2 **operators.** Left: pictorial illustration of the operators $\hat{\chi}_{3,5,7}$. Right: the expectation values $\langle \hat{\chi}_{3,5,7} \rangle$ for the four cases #1,2,3,4. The y-axis is in a log scale, and all the results are obtained by 24-site ED calculations with the field $h = 0.01 \parallel [111]$.

discrepancy as in the cases (#3,4), further analysis of the systems are necessary. For the perturbative parton analysis, one obvious way is to perform higher order perturbations. For the chirality operator method, one can try other numerical methods such as density-matrix-renormalization-group (DMRG) calculations since ED calculations with a larger system size are numerically difficult. The topological invariant is believed to be less susceptible to slight symmetry breaking from a cylinder-like lattice shape, and the chirality operator method is expected to be useful even with DMRG calculations.

Supplementay Note 9. Spin wave theory

Magnon gap and chirality. Field angle dependence of magnetically ordered phases are investigated using spin wave theories based on the linearized Holstein-Primakoff spin representation:

$$\begin{aligned} S^X &= \sqrt{\tfrac{S}{2}}(a + a^\dagger), \\ S^Y &= -i\sqrt{\tfrac{S}{2}}(a - a^\dagger), \\ S^Z &= S - a^\dagger a, \end{aligned} \tag{41}$$

where the local axis Z is defined by the classical spin configuration $\{\mathbf{S}_i^{\rm cl}\}$ of the Hamiltonian $H(\theta, \phi)$, and the other two local axes X, Y are perpendicular to the axis Z ($S = 1/2$). The boson operator a describes a local spin-flip, essentially the magnon excitation. Applying the linearized representation to the Hamiltonian $H(\theta, \phi)$ leads to the quadratic magnon Hamiltonian

$$\begin{aligned} \mathcal{H}_{\rm SW} = E_{\rm cl} &+ \sum_{\mathbf{k}} \sum_{m,n=1}^{N_{\rm s}} A_{mn}(\mathbf{k})\ a_{m,\mathbf{k}}^\dagger a_{n,\mathbf{k}} \\ &+ \sum_{\mathbf{k}} \sum_{m,n=1}^{N_{\rm s}} B_{mn}(\mathbf{k})\ a_{m,-\mathbf{k}} a_{n,\mathbf{k}} + \text{H.c.} \\ &+ \sum_{\mathbf{k}} \sum_{m=1}^{N_{\rm s}} (\mathbf{h} \cdot \mathbf{S}_m^{\rm cl}) a_{m,\mathbf{k}}^\dagger a_{m,\mathbf{k}}, \end{aligned} \tag{42}$$

where $E_{\rm cl}$ is the energy of the classical spin configuration $\{\mathbf{S}_i^{\rm cl}\}$, $N_{\rm s}$ is the number of sites in the magnetic unit cell, the subscripts m, n denote magnetic sublattices, and $\mathbf{k}$ represents momentum. The hopping amplitude $A_{mn}(\mathbf{k})$ and pairing amplitude $B_{mn}(\mathbf{k})$ are determined by the classical spin configuration. Diagonalizing the magnon Hamiltonian via Bogoliubov transformation, we obtain the magnon energy gap $\Delta_{\rm SW}$ shown in Fig. 4.

For the chirality computation, we simply use the classical spin configuration $\{\mathbf{S}_i^{\rm cl}\}$ since the chirality should be mainly determined by the local spin moments (for magnetically ordered phases). We provide the calculated chirality $\chi_{\rm SW}$ together with the magnon gap $\Delta_{\rm SW}$ in Supplementary Fig. 5.

It is important to note that there is no critical line in the magnon gap. Anisotropic spin interactions of the Kitaev and Gamma terms and the Zeeman coupling break all continuous spin rotational symmetries, so there is no gapless spin excitation. Unlike the KQSL, magnetically ordered phases do not show any resemblance/correlation between the excitation energy gap $\Delta_{\rm SW}$ and the chirality $\chi_{\rm SW}$.

Zigzag state. Based on the ED results, the zigzag antiferromagnetic state occurs in two distinct parameter regimes. One region is ($K < 0$, $\Gamma > 0$, $\Gamma' \leq 0$) in Supplementary Fig. 6a-b, and the other is ($K > 0$, $J < 0$) as shown in Fig. 7a and Supplementary Fig. 6c-d. We focus on the former case because it is believed to be more relevant to α-$RuCl_3$.

At zero field, the classical ground state manifold of $H_{KJ\Gamma\Gamma'}$ is triply degenerate consisting of x, y, z-zigzag states. These three states are related by C_3 rotation, and the name of each state implies the correlation pattern of spin moments. For instance, in the z-zigzag state spin moments are all perpendicular to the z-bond axis with being anti-aligned at each z-bond.

The degeneracy is lifted when a magnetic field is applied. The degeneracy lift pattern could be understood most intuitively at in-plane fields ($\theta = 90°$). If the magnetic field is bond-parallel, *e.g.,* aligned to the z-bond axis ($\phi = 90°$), the z-zigzag state becomes most stable compared to the other two states. In this case, the field is perpendicular to the spin moments of the z-zigzag state, which renders the z-zigzag state to develop the largest magnetization amongst the three zigzag states. By a similar mechanism, the x-zigzag state is selected near the x-bond ($\phi = 30°$) direction and the y-zigzag state

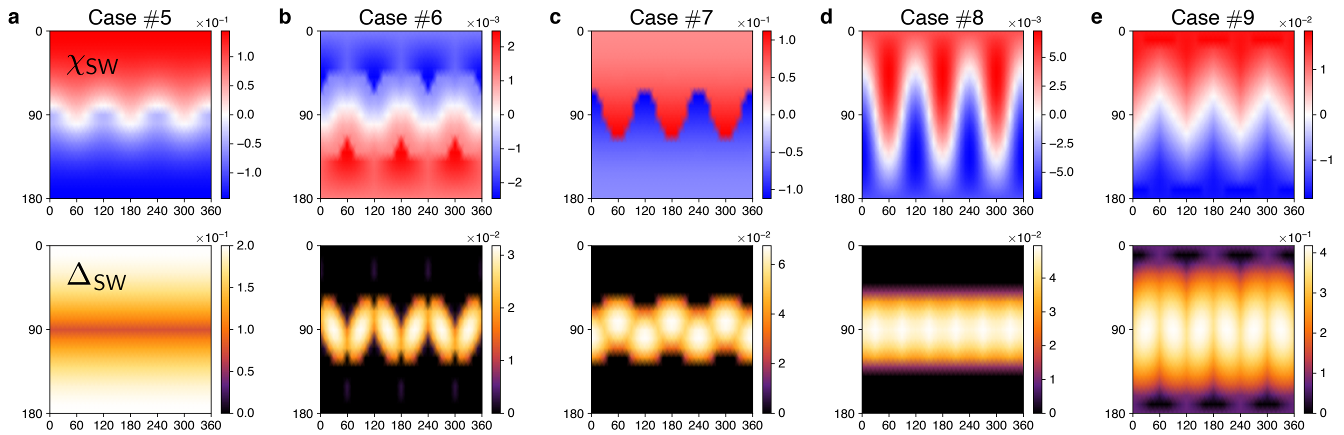

Supplementary Figure 5. **Results of the spin wave theory.** Upper and bottom: color maps of the chirality $\chi_{\text{SW}}(\mathbf{h})$, and the magnon energy gap $\Delta_{\text{SW}}(\mathbf{h})$ on the plane of the field angles (θ, ϕ) (horizontal axis: ϕ [°], vertical axis: θ [°]). The parameter sets used in the five cases (#5 $\sim$ 9) are listed in Table 1. The magnetic field strength is fixed by $h = 0.1$ in the cases #5 $\sim$ 8 and $h = 0.06$ in the case #9. The black regions in the magnon gap indicate the regions where each magnetic order becomes unstable by magnon condensation.

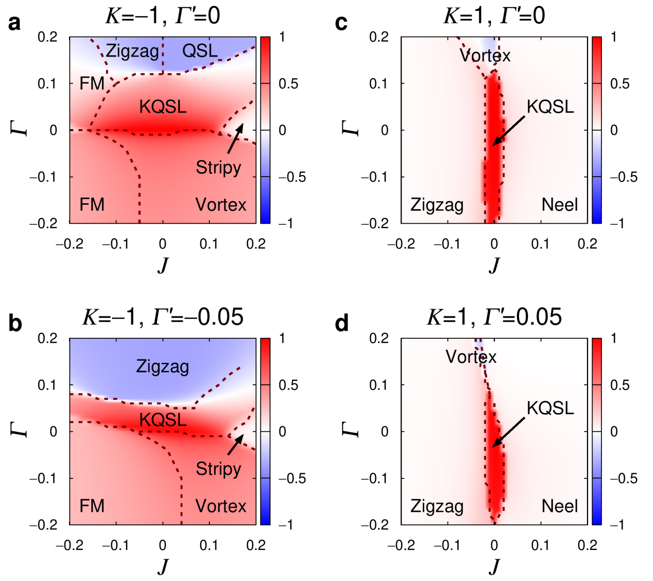

Supplementary Figure 6. $\boldsymbol{\Gamma'}$ **effects on the phases of the** $\boldsymbol{K}$**-**$\boldsymbol{J}$**-**$\boldsymbol{\Gamma}$**-**$\boldsymbol{\Gamma'}$ **model.**

is selected near the y-bond ($\phi = 150°$) direction. From this analysis, we gain the insight that the zigzag phase becomes most stable at the *bond-parallel* field directions $\phi = 30° + n \cdot 60°$ $(n = 0, 1, 2, 3, 4, 5)$. It is corroborated with the largest size of magnon gap Δ_{magnon} found at the bond-parallel directions (Fig. 5b).

We have checked the same pattern of field angle dependence in the J-K-Γ-J_3 model proposed for α-$RuCl_3$ [7, 8] as well.

MES	$\frac{\zeta}{\pi/2}$	$\frac{\eta}{\pi/2}$	$\frac{\alpha}{2\pi}$	$\frac{\beta}{2\pi}$	S_A
$\lvert\Psi_1^{\text{MES-I}}\rangle$	7/10	3/10	5/12	7/12	2.22
$\lvert\Psi_2^{\text{MES-I}}\rangle$	5/10	10/10	0/12	8/12	2.36
$\lvert\Psi_3^{\text{MES-I}}\rangle$	6/10	5/10	11/12	1/12	3.15
$\lvert\Psi_1^{\text{MES-II}}\rangle$	7/10	3/10	1/12	11/12	2.22
$\lvert\Psi_2^{\text{MES-II}}\rangle$	5/10	10/10	0/12	4/12	2.36
$\lvert\Psi_3^{\text{MES-II}}\rangle$	6/10	5/10	7/12	5/12	3.15

Supplementary Table 3. **Minimally entangled states.** The MES are obtained by searching on a $10 \times 10 \times 12 \times 12$ uniform grid of $(\zeta, \eta, \alpha, \beta)$ for the case #4 of Table 1, $(K, J, \Gamma, \Gamma') = (-1, 0.08, 0.01, 0.05)$ & $h = 0.01 \parallel [111]$.

Supplementay Note 10. Topological degeneracy and modular matrix

The non-abelian KQSL state has threefold ground state degeneracy on a torus geometry due to the Ising anyon topological order [2, 9]. In Supplementary Fig. 7, we demonstrate the degeneracy for a few selected field directions. It is clearly shown that the lowest three states (degeneracy slightly lifted due to the finite size effect) are well separated from the other excited states for small h. Furthermore, it is verified that those quasi-degenerate states share qualitatively same bulk properties such as the expectation value of flux operator $\langle \hat{W}_p \rangle$, spin structure factor $S(\mathbf{q})$, and also chirality $\chi(\mathbf{h})$.

By using the topological degenerate states, we may extract the modular $\mathcal{S}$ matrix containing the information of quasiparticles' statistics and fusion rules. It is achieved by finding the so called minimally entangled states (MES) in the subspace of the quasi-degenerate

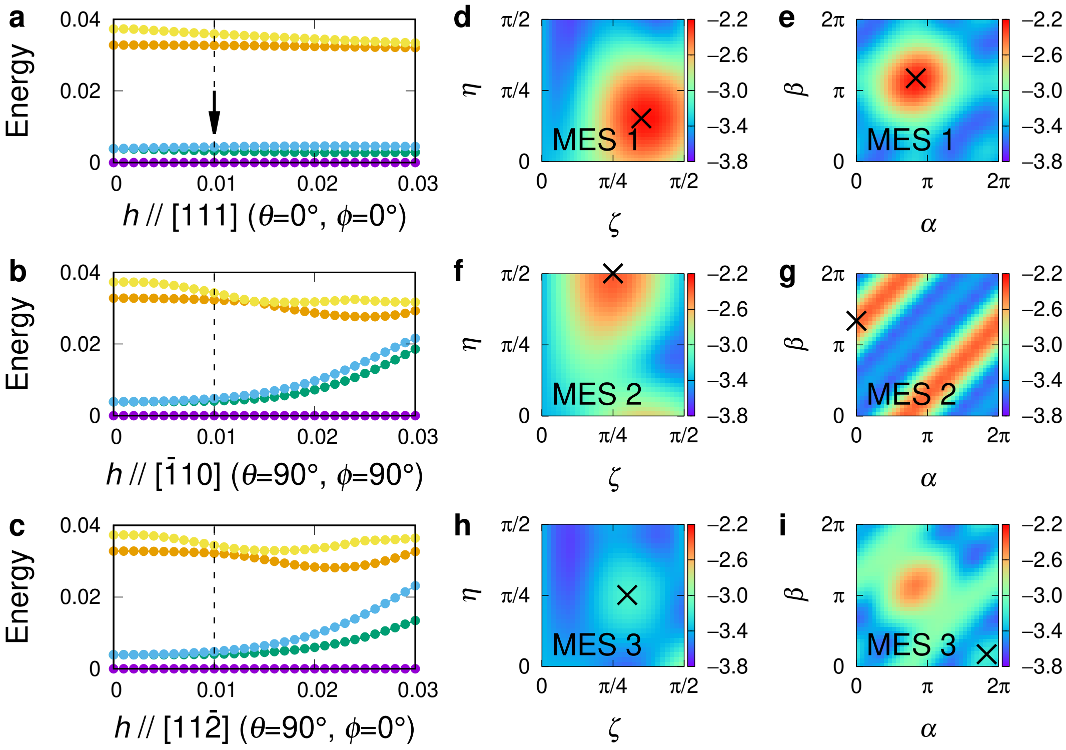

Supplementary Figure 7. **Topological degeneracy and entanglement entropy. a-c** Evolution of the energy spectrum for three different field directions. **d-i** Color maps of the entanglement entropy $S_A(\zeta, \eta, \alpha, \beta)$ near the states $\{|\Psi_m^{\mathrm{MES-I}}\rangle\}_{m=1}^3$ in Supplementary Table 3. In each map, the entropy is plotted with a reversed sign, $-S_A$, which reveals the MES through local maxima. The cross marks denote the location of $|\Psi_1^{\mathrm{MES-I}}\rangle$ in **d-e**, $|\Psi_2^{\mathrm{MES-I}}\rangle$ in **f-g**, and $|\Psi_3^{\mathrm{MES-I}}\rangle$ in **h-i**. All the results are obtained for the case #4 of Table 1, $(K, J, \Gamma, \Gamma') = (-1, 0.08, 0.01, 0.05)$, and the computations of entanglement entropy and MES are conducted for the magnetic field $h = 0.01 \parallel [111]$ (marked by an arrow in **a**).

states [10, 11]:

$$|\Psi\rangle = \sum_{n=1}^{3} z_n |\Psi_n\rangle, \tag{43}$$

where $|\Psi_{1,2,3}\rangle$ are the three quasi-degenerate states, and the complex coefficients $z_{1,2,3}$ are parametrized by

$$z_1 = \sin\zeta \cos\eta, \tag{44}$$

$$z_2 = \sin\zeta \sin\eta e^{i\alpha}, \tag{45}$$

$$z_3 = \cos\zeta e^{i\beta}, \tag{46}$$

with the four angles, $\zeta, \eta \in [0, \pi/2]$ and $\alpha, \beta \in [0, 2\pi]$.

To construct the minimally entangled states, we consider two noncontractable bipartitions on the torus geometry (cut I and cut II) [10, 11]. Then for each bipartition we compute the entanglement entropy

$$S_A = -\log \mathrm{Tr}\rho_A^2 \tag{47}$$

where ρ_A is the reduced density matrix $\rho_A \equiv \mathrm{Tr}_B|\Psi\rangle\langle\Psi|$ traced over the partition B of the torus. The minimally entangled states $\{|\Psi_n^{\mathrm{MES}}\rangle\}_{n=1}^3$ correspond to the local minima of S_A in the parameter space of $(\zeta, \eta, \alpha, \beta)$. We list the resulting MES in Supplementary Table 3, and illustrate the entanglement entropy $S_A(\zeta, \eta, \alpha, \beta)$ near the MES points in Supplementary Fig. 7d-i. After an appropriate U(1) transformation in each of the MES, the inner products between the two sets of the MES

$$\mathcal{S}_{mn} = \langle \Psi_m^{\mathrm{MES-I}} | \Psi_n^{\mathrm{MES-II}} \rangle \tag{48}$$

yield the modular $\mathcal{S}$ matrix of the Ising topological field theory in Eq. (7).

Case	K	J	Γ	Γ'	Phase	Supplementary figure
#10	-1	0	0	0.0005	KQSL	8a
#11	-1	0	0.01	-0.01	KQSL	8b
#12	1	0	0	0	KQSL	8c
#13	1	0	0.05	0	KQSL	8d

Supplementary Table 4. **Additional parameter sets for exact diagonalization.**

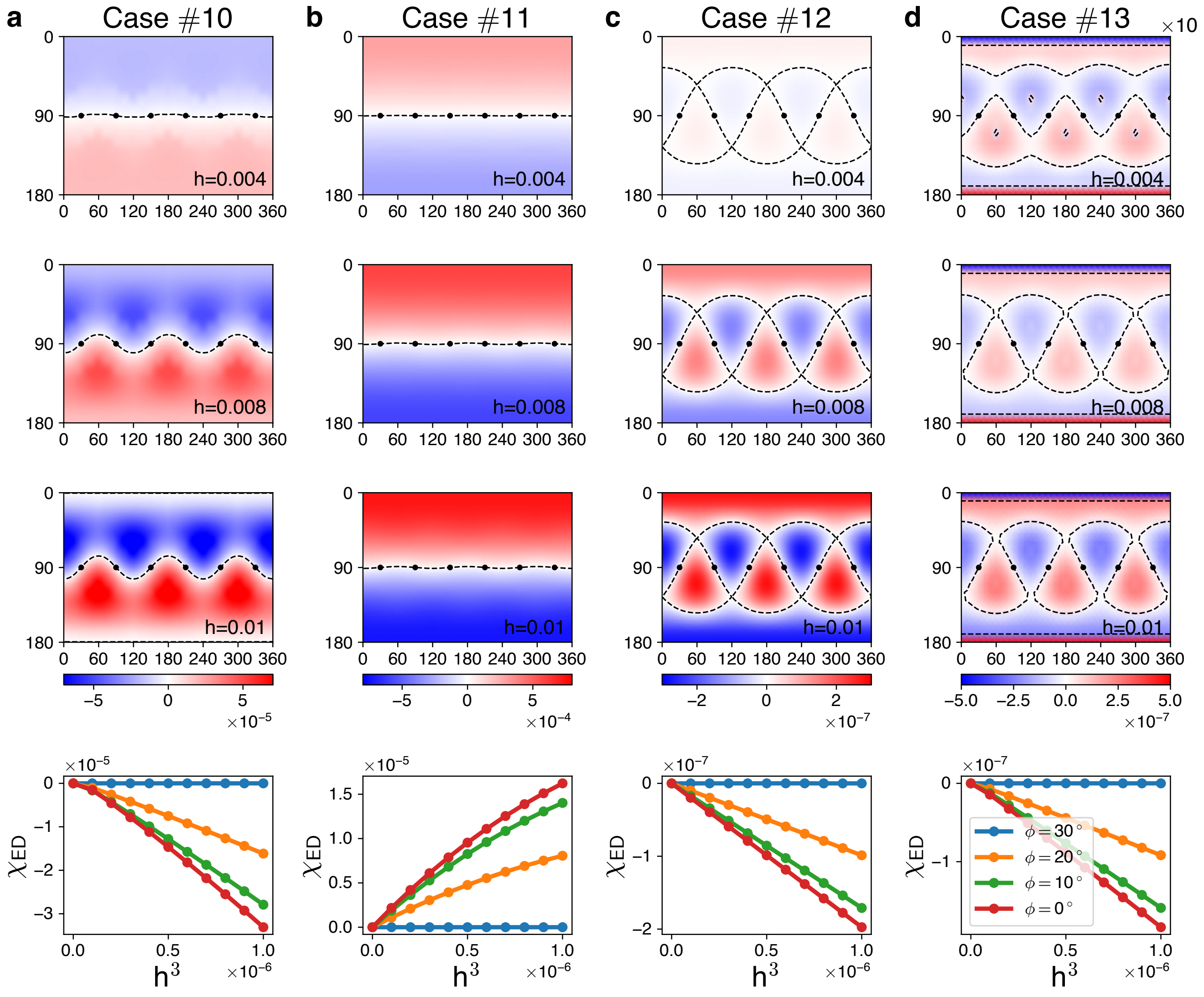

Supplementary Figure 8. **Additional results of the chirality for the non-abelian KQSL.** Top three: color maps of the chirality $\chi_{\mathrm{ED}}(\mathbf{h})$ on the plane of the field angles (θ, ϕ) for the magnetic field strength $h = 0.004,\ 0.008,\ 0.01$ (horizontal axis: ϕ [°], vertical axis: θ [°]). The dashed lines highlight the zero lines $\chi_{\mathrm{ED}}(\mathbf{h}) = 0$, and the black dots mark the bond directions. Bottom: $\chi_{\mathrm{ED}}(\mathbf{h})$ as a function of h^3 for the in-plane fields ($\theta = 90°, \phi = 0°, 10°, 20°, 30°$), illustrating the universality of the h^3 behavior in the KQSL. The parameter sets used in the four cases (#10 $\sim$ 13) are listed in Supplementary Table 4.

Supplementay Note 11. Additional results of the chirality χ_{ED}

Supplementary Fig. 8 presents additional results of the chirality χ_{ED}, obtained for the parameter sets in Supplementary Table 4. The first three cases #10 $\sim$ 12 are well understood by the competition between the h-linear term $(h_x + h_y + h_z)$ and the h-cubic term $(h_x h_y h_z)$. By contrast, the case #13 shows distinguished field evolution behaviors from the other cases. For instance, high intensity peaks of χ_{ED} are observed near the two poles $\theta = 0°,\ 180°$, enclosed by additional critical lines. These features are attributed to effects of other h-cubic terms such as $F_3'(\mathbf{h})$ and $F_3''(\mathbf{h})$ in Supplementary Table 2.

The cubic dependence for in-plane fields is confirmed in most of the cases. We find a slight deviation from the cubic dependence in the case #10 near the zero field. To confirm the cubic dependence in ED calculations, it is important to make sure that topological degeneracy is lifted by a small but finite energy gap, eliminating the degeneracy in the ground state (as in Supplementary Fig. 7a-c).

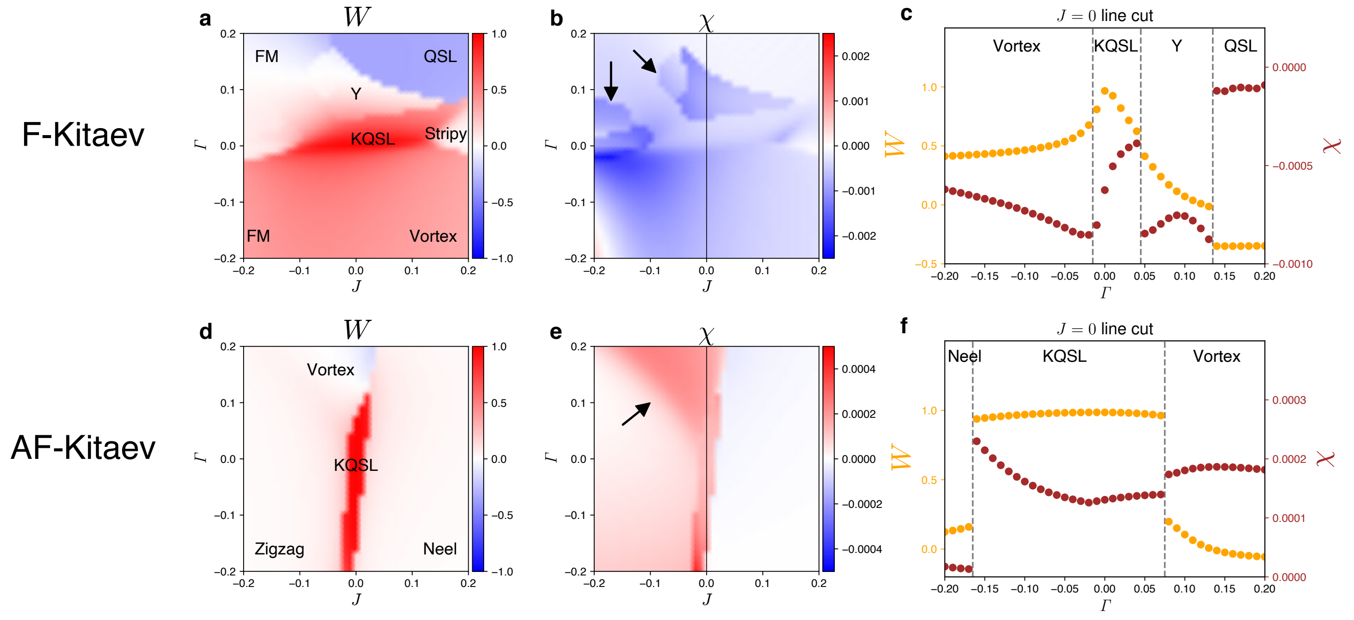

Supplementary Figure 9. **Distinct phase boundaries in the chirality.** **a-c** ED results for $K = -1$, $\Gamma' = 0.05$, $h = 0.01 \parallel [111]$ with the flux W $(= \langle \hat{W}_p \rangle)$, the chirality χ $(= \langle \hat{\chi}_p \rangle)$, and a comparison of W and χ along the $J = 0$ line cut. **d-f** ED results for $K = 1$, $\Gamma' = -0.05$, $h = 0.01 \parallel [111]$ with the flux W, the chirality χ, and a comparison of W and χ along the $J = 0$ line cut.

Supplementay Note 12. Identification of phase boundaries by the chirality

Distinct phase boundaries under magnetic field can be identified clearly by the chirality operator. We demonstrate this by conducting additional ED calculations as shown in Supplementary Fig. 9. We find that the phase boundaries revealed by the plaquette operator $(\hat{W}_p)$ are equally well captured by the chirality operator $(\hat{\chi}_p)$. In some cases, the chirality operator works better than the plaquette operator as shown in Supplementary Fig. 9b,e (marked by arrows). This shows that the chirality is also useful for the identification of distinct phase boundaries of the Kitaev system.

[1] Winkler, R., Papadakis, S., De Poortere, E. & Shayegan, M. *Spin-Orbit Coupling in Two-Dimensional Electron and Hole Systems*, vol. 41 (Springer, 2003).

[2] Kitaev, A. Anyons in an exactly solved model and beyond. *Ann. Phys. (Amsterdam)* **321**, 2 (2006).

[3] Takikawa, D. & Fujimoto, S. Impact of off-diagonal exchange interactions on the Kitaev spin-liquid state of α-$RuCl_3$. *Phys. Rev. B* **99**, 224409 (2019).

[4] Baskaran, G., Mandal, S. & Shankar, R. Exact results for spin dynamics and fractionalization in the Kitaev model. *Phys. Rev. Lett.* **98**, 247201 (2007).

[5] Han, J. H. & Lee, H. Spin chirality and Hall-like transport phenomena of spin excitations. *J. Phys. Soc. Jpn.* **86**, 011007 (2017).

[6] Nasu, J., Yoshitake, J. & Motome, Y. Thermal transport in the Kitaev model. *Phys. Rev. Lett.* **119**, 127204 (2017).

[7] Winter, S. M. *et al.* Breakdown of magnons in a strongly spin-orbital coupled magnet. *Nat. Commun.* **8**, 1152 (2017).

[8] Winter, S. M., Riedl, K., Kaib, D., Coldea, R. & Valentí, R. Probing α-$RuCl_3$ beyond magnetic order: Effects of temperature and magnetic field. *Phys. Rev. Lett.* **120**, 077203 (2018).

[9] Kells, G., Slingerland, J. K. & Vala, J. Description of kitaev's honeycomb model with toric-code stabilizers. *Phys. Rev. B* **80**, 125415 (2009).

[10] Zhang, Y., Grover, T., Turner, A., Oshikawa, M. & Vishwanath, A. Quasiparticle statistics and braiding from ground-state entanglement. *Phys. Rev. B* **85**, 235151 (2012).

[11] Zhu, W., Gong, S. S., Haldane, F. D. M. & Sheng, D. N. Identifying non-abelian topological order through minimal entangled states. *Phys. Rev. Lett.* **112**, 096803 (2014).
